# Session-specific effects of the Metacognitive Group Training for Obsessive–Compulsive Disorder: significant results for thought control

**DOI:** 10.1038/s41598-020-73122-z

**Published:** 2020-10-20

**Authors:** Franziska Miegel, Barbara Cludius, Birgit Hottenrott, Cüneyt Demiralay, Lena Jelinek

**Affiliations:** 1 Department of Psychiatry and Psychotherapy, University Medical Center Hamburg-Eppendorf, Martinistrasse 52, 20246 Hamburg, Germany; 2 LMU Munich, Department of Psychology, Clinical Psychology and Psychotherapy, Munich, Germany

**Keywords:** Human behaviour, Obsessive compulsive disorder

## Abstract

The investigation of the session-specific effects is central for the understanding of psychological interventions. For the present study, we investigated the session-specific effects of the Metacognitive Group Training for Obsessive–Compulsive Disorder (MCT-OCD), which was revised based on data of a pilot study. Thirty-four outpatients with OCD participated in the MCT-OCD once a week over 8 weeks. Different metacognitive beliefs (e.g., thought control) and cognitive beliefs (e.g., intolerance of uncertainty), OC symptoms, as well as associated comorbid symptoms were assessed before and after each session. Linear mixed effects models showed that patients’ obsessions and compulsions, thought control, the belief of being well informed about the disorder, and action fusion improved over the course of the training. The only session-specific effect emerged for thought control, which improved immediately after the respective module. We were able to replicate the findings of the pilot study and thus corroborate the session-specific effect of the module targeting thought control. Moreover, we generated information on the mode of action of the individual modules of the MCT-OCD that allows a more in-depth evaluation of the intervention. Notably, we were able to eliminate the adverse effects of the pilot version of the MCT-OCD.

**Trial Registration**: German Clinical Trials Register (Deutsches Register Klinischer Studien [DRKS]; DRKS-ID: DRKS00013539; registration date: 22/02/2018).

## Introduction

The efficacy of a psychological intervention is most commonly investigated by comparing symptom severity before and after an intervention^1,2^ that often continues for weeks and encompasses multiple sessions targeting different topics. In this way, the “success” of an intervention is determined by the decrease in symptoms. Of course, this represents valid and important information, and pre- versus post-intervention effects represent the primary outcome of the research. However, a major limitation is that these kinds of analyses do not allow consideration of confounders (e.g., other simultaneous treatments) and thus symptom reduction cannot be attributed to the intervention alone^3^. Researchers use control groups to try to circumvent these problems. Nevertheless, even though we imagine we can rely on our finding that a certain intervention reduced patients’ symptoms, we still do not know whether the components of an intervention change patients’ symptoms and cognitive biases the way we planned to address them. Knowing this might help us to identify the most relevant parts and give us the opportunity to streamline the interventions^4,5^.

For patients with obsessive–compulsive disorder (OCD), the most effective psychological therapy is cognitive-behavioral therapy (CBT) with exposure and response prevention (ERP)^6^. However, as CBT with ERP requires administration by trained professionals, which are rare, patients with OCD wait on average five months until receiving treatment^ 7^. There is a need for treatments that are (1) highly standardized and thus can be easily administered (even by less experienced professionals) and disseminated and (2) facilitate session-specific evaluation, which would allow an optimal tailoring of the intervention in the long-run. Therefore, our research group developed the Metacognitive Group Training for OCD (MCT-OCD), which fulfills the above-mentioned criteria. Originally, the MCT-OCD was derived from the Metacognitive Training for Psychosis (MCT)^8^.

The MCT-OCD targets in several modules (meta)cognitive beliefs that were identified by the Obsessive–Compulsive Cognitions Working Group (OCCWG)^9–11^. These targeted (meta)cognitive beliefs are perfectionism (module #2), intolerance of uncertainty (module #3), action fusion (module #4), control of thoughts (module #5), overestimation of threat (module #6), and inflated sense of responsibility (module #7). Module #1 (false assumptions about OCD) addresses patients’ concerns regarding specific fears (e.g., “I cannot do anything to reduce my symptoms”). Module #8 (biased attention/biased cognitive networks) expands the content by addressing patients’ biased attention to OCD-relevant stimuli and the patients’ biased cognitive networks.

The pilot version of the MCT-OCD was highly accepted by patients^12^. In a subsequent randomized controlled trial the revised version of the MCT-OCD was compared to a wait-list control group with access to care as usual^13^.

The session-specific effects of the pilot version of the MCT-OCD have been investigated recently^14^ in order to determine whether the modules specifically improve the (meta)cognitive belief that is targeted in the respective module. Consistent with our hypotheses, we found that patients’ thought monitoring, thought control, obsessions, and compulsions improved over the duration of the treatment. Notably, patients’ thought control improved most after the respective module, showing a very specific effect of this module. As the pre- and post-questionnaires for the session-specific evaluation did not cover all of the modules’ contents (i.e., the questionnaires did not, for example, assess perfectionism so that the session-specific effect of the perfectionism module could not be evaluated), we were not able to draw specific within-session conclusion from the other modules. Therefore, the questionnaires were revised to cover all topics of the modules^14^. Additionally, the impact of the modules on patients’ mood was divergent in the previous study^14^. Compared to all the other modules, the module about overestimation of threat and exaggerated sense of responsibility worsened patients’ mood, whereas the module on perfectionism and intolerance of uncertainty enhanced patients’ mood, immediately after the module. Patients’ mood, however, did not improve over the course of the treatment. Thus, the results on patients’ mood were heterogeneous and rather unsatisfactory. Therefore, the layout of the slide-based presentation was revised in order to make it more playful, and the module on overestimation of threat and exaggerated sense of responsibility was also revised by adding more humorous elements to the module to address the results regarding patients’ mood. Moreover, in contrast to the pilot study in which two (meta)cognitive beliefs were addressed in one session, the number of modules was increased, and each module now covers only one (meta)cognitive belief. This allows a much more specific evaluation of the sessions.

The current study builds upon the pilot study^14^ and extends the previous findings by investigating the session-specific effects of all modules more specifically in order to determine whether the modules specifically improve the (meta)cognitive belief targeted in the respective module. This may help with a future revision of the MCT-OCD in order to make the intervention as effective as possible and to identify if the modules change the addressed (meta)cognitive belief. To achieve this, we evaluated patients’ metacognitive (e.g., thought control) and cognitive beliefs (e.g., overestimation of threat), OC symptoms (e.g., obsessions), and associated comorbid symptoms (e.g., depression) before and after each MCT-OCD session by calculating linear mixed-effects models (LMMs). We hypothesized that all investigated (meta)cognitive beliefs, OC symptoms, and associated comorbidities would decrease over the period of the intervention. Moreover, we assumed that the (meta)cognitive belief (e.g., overestimation of threat, thought control) that is targeted in a particular session (e.g., module #6, module #5) would be reduced immediately after the session (within-session effect) and one week after the session (between-session effect).

## Results

See Table 1 for the demographic and psychopathological data of the sample. Patients participated in six modules on average (*M* = 5.82, *SD* = 1.66), with a range from two to eight. The main current comorbid disorders as verified by the M.I.N.I. were depression (17.8%) and anxiety disorders (agoraphobia: 17.6%; generalized anxiety disorder: 14.7%; social anxiety disorder: 11.8%; panic disorder: 8.8%).

**Table 1:** Demographic and psychopathological data: mean (*M*) and standard deviation (*SD*) and frequency (*n*) and percent (%).

	Total (*N* = 34)	
	*M* or *n*	*SD* or %
Age (years)	37.53	10.06
Sex (m/f)	16/18	47.1/52.9
Formal education (years)	11.94	1.30
Illness duration (years)	21.01	10.91
Age at OCD onset (years)	16.50	10.32
Current outpatient treatment	22	64.7
Y-BOCS total score	20.21	5.68
HDRS total score	8.76	6.34
**Medication intake**		
Antidepressant	13	38.2
Antipsychotic	0	0
Combination	1	2.9
None	20	58.8

*HDRS* Hamilton Depression Rating Scale, *Y-BOCS* Yale-Brown Obsessive Compulsive Scale.

For the treatment effect over time the random intercept model was superior for the majority of the variables, with the exception of both variables on the belief of being well informed about the disorder, reduced intolerance of uncertainty 2, perfectionism 2 (see Supplementary material A for a detailed description of all items), both variables on action fusion, overestimation of threat 1, inflated sense of responsibility 2, and biased attention. For those models, the random intercept, random slope model was a better fit. The random intercept model showed an improvement in thought control (*b* =  − 0.10, 95% CI [− 0.15, − 0.05], *p* < .001), obsessions (*b* =  − 0.07, 95% CI [− 0.13, − 0.01], *p* = .023), and compulsions (*b* =  − 0.07, 95% CI [− 0.12, − 0.03], *p* = .002) over time. The random intercept, random slope model showed an improvement in the belief of being well informed about the disorder 1 (*b* = 0.08, 95% CI [0.02, 0.14], *p* = .012), action fusion 1 (*b* =  − 0.08, 95% [CI − 0.14, − 0.02], *p* = .010), and action fusion 2 (*b* =  − 0.07, 95% CI [− 0.13, − 0.02], *p* = .011). All other variables remained insignificant (|*b*|≤ 0.04, *p* ≥ .069).

For most within-session analyses, the random intercept model was used (see Table 2). In accordance with our hypotheses, the calculations demonstrated that patients’ thought control reduced the most after module #5 (control of thoughts; with a small to medium effect of *d* = 0.42) compared to the average score on thought control after all other modules. The change in all other variables remained insignificant (see Table 2).

**Table 2:** Results of the linear mixed-effects model for all modules, within-session changes, presented as beta value for fixed effect (*B*).

Module	Dependent variable																		
	DV1	DV2	DV3	DV4	DV5	DV6	DV7	DV8	DV9	DV10	DV11	DV12	DV13	DV14	DV15	DV16	DV17	DV18	DV19
Range (intercept)	1.18–1.39	1.55–1.64	1.24–1.44	0.63–0.74	1.31–1.41	2.11–2.24	2.25–2.49	1.16–1.57	1.44–1.51	1.65–1.76	1.31–1.71	1.02–1.05	1.28–1.35	1.35–1.49	1.49–1.59	0.57–0.63	0.60–0.63	1.62–1.70	0.76–0.69
Module 1	− 0.07	0.16	− 011	− 0.02	− 0.10	− 0.03	− 0.09	− 0.19	− 0.15	− 0.15	0.04	− 0.05	0.07	0.15	− 0.19	0.04	− 0.10	0.19	0.11
Module 2	0.08	0.25	0.19	0.14	− 0.21	− 0.53	− 0.04	− 0.07	0.09	0.14	0.13	− 0.13	0.09	− 0.12	0.29	− 0.13	− 0.07	0.24	0.02
Module 3	− 0.22	− 0.28	− 0.37	0.01	− 0.27	0.15	0.13	− 0.17	0.02	0.04	0.04	0.06	0.07	0.08	0.06	− 0.06	− 0.02	− 0.10	− 0.15
Module 4	− 0.05	0.08	0.07	− 0.05	− 0.06	0.15	− 0.07	− 0.13	0.11	− 0.12	0.01	0.07	− 0.23	− 0.26	0.15	0.18	0.15	− 0.12	0.13
Module 5	− 0.09	− 0.46*	0.06	− 0.02	0.37	0.14	− 0.00	0.05	− 0.19	− 0.12	− 0.19	− 0.19	− 0.24	− 0.17	0.00	− 0.12	0.02	− 0.01	− 0.14
Module 6	0.13	0.18	0.03	− 0.12	0.05	0.05	− 0.03	0.28	0.04	− 0.19	0.03	0.07	− 0.06	− 0.00	− 0.24	− 0.03	− 0.00	− 0.31	− 0.07
Module 7	0.09	0.11	− 0.07	− 0.04	− 0.02	0.07	0.01	0.07	− 0.03	0.24	0.10	0.09	0.11	0.02	− 0.14	0.15	0.09	0.02	0.01
Module 8	0.16	0.00	0.23	0.09	0.16	− 0.06	0.10	0.10	0.12	0.20	− 0.06	0.08	0.18	0.25	0.11	− 0.03	− 0.13	0.14	0.11
**Random parts**																			
Time	0.31–0.33	0.45–0.47	0.44–0.49	0.26–0.32	0.51–0.52	0.25–0.33	0.15–0.16	0.29–0.37	0.37	0.40–0.44	0.19–0.24	0.26	0.29–0.30	0.37	0.32–0.33	0.37–0.38	0.26–0.34	0.36–0.37	0.38–0.43
Patients	0.33–0.35	0.31–0.32	0.40–0.41	0.21–0.22	0.18–0.19	0.01–0.03	0.14–0.23	0.15–0.27	0.43–0.45	0.19–0.25	0.14–0.18	0.34–0.36	0.49–0.50	0.30–0.33	0.34–0.37	0.05–0.06	0.09–0.13	0.18–0.19	0.01–0.02
ICC	0.50–0.52	0.40–0.41	0.45–0.51	0.40–0.50	0.26–0.27	0.04–0.20	0.54–0.56	0.29–0.51	0.54–0.55	0.35–0.41	0.37–0.48	0.57–0.58	0.62–0.63	0.45–0.47	0.51–0.53	0.12–0.14	0.20–0.38	0.33–0.34	0.03–0.15
Nid	34	34	34	34	34	34	34	34	34	34	34	34	34	34	34	34	34	34	34
Observations (*N*)	198	193	196	192	196	193	198	194	198	193	197	192	197	192	196	191	198	191	198

DV1 = Thought monitoring; DV2 = Thought control; DV3 = Obsessions; DV4 = Compulsions; DV5 = Mood; DV6 = Belief of being well informed about the disorder 1, DV7 = Belief of being well informed about the disorder 2; DV8 = Perfectionism 1; DV9 = Perfectionism 2; DV10 = Intolerance of uncertainty 1; DV11 = Intolerance of uncertainty 2; DV12 = Action fusion 1; DV13 = Action fusion 2; DV14 = Overestimation of threat 1; DV15 = Overestimation of threat 2; DV16 = Inflated sense of responsibility 1; DV 17 = Inflated sense of responsibility 2; DV18 = Biased attention; DV19 = Biased cognitive networks. Module 1 = False assumptions about OCD; Module 2 = Perfectionism; Module 3 = Intolerance of uncertainty; Module 4 = Action fusion; Module 5 = Control of thoughts; Module 6 = Overestimation of threat; Module 7 = Inflated sense of responsibility; Module 8 = Biased attention/biased cognitive networks. For most analyses, the random intercept model was used. For DV1 (module 4), DV3 (module 2), DV4 (module 1), DV6 (modules 1 and 2), DV7 (modules 5 and 8), DV8 (module 1), DV10 (module 1), DV11 (modules 7 and 8), DV17 (module 1), and DV19 (module 5), the random intercept, random slope module was used.

*ICC* intraclass correlations.

*p < .05.

For most between-session analyses, the random intercept model was used (see Table 3). Patients’ intolerance of uncertainty 2 worsened more one week after module #1 (false assumptions about OCD; with a medium effect size of *d* = 0.52) compared to the average score on intolerance of uncertainty 2 after all other modules. All other variables remained insignificant (see Table 3).

**Table 3:** Results of the linear mixed-effects model for all modules, between-session changes, presented as beta value for fixed effect (*B*).

Module	Dependent variable																		
	DV1	DV2	DV3	DV4	DV5	DV6	DV7	DV8	DV9	DV10	DV11	DV12	DV13	DV14	DV15	DV16	DV17	DV18	DV19
Range intercept)	1.40–1.49	2.26–2.58	1.96–2.08	1.35–1.42	2.39–2.48	2.36–2.57	1.87–1.94	0.81–0.92	1.02–1.11	2.66–2.90	3.27–3.61	0.46–0.53	0.78–0.94	1.17–1.44	1.14–1.32	0.96–1.46	0.67–0.82	1.88–2.02	2.23–2.54
Module 1	− 0.04	0.04	0.26	0.14	0.19	0.11	0.17	0.49	0.27	0.24	0.34*	0.16	0.38	0.12	0.09	− 0.13	0.02	− 0.01	0.19
Module 2	0.15	0.16	− 0.20	− 0.40	0.08	0.18	− 0.17	− 0.17	0.03	− 0.14	− 0.14	0.15	− 0.09	0.09	− 0.38	0.14	0.33	− 0.18	− 0.03
Module 3	0.00	− 0.05	0.36	0.23	0.33	0.01	− 0.07	− 0.08	− 0.21	− 0.09	− 0.03	− 0.05	0.19	− 0.08	− 0.14	0.18	0.05	0.23	0.16
Module 4	− 0.05	− 0.00	− 0.04	0.02	− 0.04	− 0.12	− 0.02	0.00	0.09	− 0.08	− 0.17	− 0.09	0.14	0.01	− 0.06	− 0.21	− 0.43	0.04	0.11
Module 5	0.05	0.19	0.16	0.23	0.27	− 0.02	0.16	− 0.18	− 0.01	0.10	0.17	0.01	− 0.30	− 0.03	0.07	0.15	0.25	0.15	0.04
Module 6	0.14	− 0.20	− 0.33	− 0.02	− 0.26	0.09	0.18	0.202	− 0.24	− 0.04	0.05	− 0.29	− 0.26	0.03	0.17	− 0.37	− 0.32	− 0.24	− 0.40
Module 7	− 0.25	0.12	− 0.21	− 0.04	− 0.14	− 0.12	− 0.20	0.03	− 0.05	0.04	0.01	0.04	− 0.16	− 0.28	0.24	− 0.05	− 0.01	0.11	0.11
Module 8	− 0.02	− 0.17	0.01	− 0.19	0.15	− 0.08	− 0.06	− 0.06	0.10	− 0.04	− 0.19	0.10	0.10	0.19	0.05	0.25	0.10	− 0.21	− 0.14
**Random parts**																			
Time	0.59–0.66	0.61–0.74	1.02–1.03	0.61–0.63	0.84–0.85	0.29–0.35	0.39–0.40	0.54–0.64	0.73–0.85	0.36–0.41	0.14–0.17	0.54–0.55	0.50–0.60	0.44–0.56	0.29–0.30	0.48–0.64	0.56–0.58	0.45–0.55	0.44–0.46
Patients	0.10–0.11	0.28–0.46	0.03–0.05	0.20–0.24	0.10–0.12	0.06–0.07	0.01–0.02	0.00–0.01	0.00–0.06	0.36–0.49	0.15–0.23	0.00–0.00	0.00–0.01	0.11–0.27	0.13–0.19	0.06–0.30	0.03–0.05	0.16–0.22	0.07–0.12
ICC	0.13–0.21	0.28–0.43	0.02–0.05	0.24–0.28	0.11–0.13	0.16–0.32	0.04–0.05	0.00–0.00	0.00–0.14	0.49–0.55	0.46–0.49	0.00–0.00	0.01–0.17	0.16–0.38	0.39–0.42	0.09–0.37	0.05–0.08	0.23–0.37	0.13–0.23
Nid	33	33	33	33	33	33	33	33	33	33	33	33	33	33	33	33	33	33	33
Observations (*N*)	154	149	152	148	152	148	154	149	154	148	154	148	153	148	152	146	154	146	154

DV1 = Thought monitoring; DV2 = Thought Control; DV3 = Obsessions; DV4 = Compulsions; DV5 = Mood; DV6 = Belief of being well informed about the disorder 1; DV7 = Belief of being well informed about the disorder 2; DV8 = Perfectionism 1; DV9 = Perfectionism 2; DV10 = Intolerance of uncertainty 1; DV11 = Intolerance of uncertainty 2; DV12 = Action fusion 1; DV13 = Action fusion 2; DV14 = Overestimation of threat 1; DV15 = Overestimation of threat 2; DV16 = Inflated sense of responsibility 1; DV17 = Inflated sense of responsibility 2; DV18 = Biased attention; DV19 = Biased cognitive networks. Module 1 = False assumptions about OCD; Module 2 = Perfectionism; Module 3 = Intolerance of uncertainty; Module 4 = Action fusion; Module 5 = Control of thoughts; Module 6 = Overestimation of threat; Module 7 = Inflated sense of responsibility; Module 8 = Biased attention/biased cognitive networks. For DV1 (module 3), DV2 (module 8), DV6 (module 1), DV8 (module 8), DV9 (module 2), DV10 (modules 3 and 4), DV11 (module 8), DV13 (module 3), DV14 (module 8), DV15 (module 5), DV16 (module 8), and DV18 (module 2) the random intercept, random slope module was used.

*ICC* intraclass correlations.

*p < .007 (Bonferroni correction).

## Discussion

Based on the results of the pilot study^14^, we aimed to investigate the session-specific effects of the revised metacognitive training for patients with OCD (MCT-OCD) using expanded pre- and post-questionnaires. In accordance with the results of the uncontrolled pilot study on the session-specific effects of the MCT-OCD^14^ and our hypotheses, patients’ thought control, obsessions, and compulsions improved over the duration of the treatment. In line with our hypotheses, patients’ belief of being well informed about the disorder and patients’ degree of action fusion also improved over treatment. The other (meta)cognitive beliefs and associated comorbid symptoms did not improve over the duration of the treatment.

Regarding session-specific effects, we were able to replicate and specify the results of the pilot study^14^ showing that patients’ control of thoughts improved most after the relevant module (module #5). As the pilot version of the MCT-OCD comprised two (meta)cognitive beliefs per module (e.g., thought-action fusion/control of thoughts), this was not self-evident. Hence, the present study increased the clarity and strengthened the validity of the finding. However, none of the other modules improved the specific (meta)cognitive belief that was targeted in the respective module. Several potential causes may have contributed to this result: (1) the items did not assess the (meta)cognitive belief adequately and thus need to be revised and extended, (2) the modules did not address the (meta)cognitive beliefs as specifically as necessary and need to be revised, (3) it takes longer for certain (meta)cognitive beliefs to change, or (4) the (meta)cognitive beliefs that are addressed by the modules did not change the respective (meta)cognitive belief. The reasons for the insignificant within-session findings of the present study cannot fully be determined on the basis of our data. Future studies need to assess variables apart from the (meta)cognitive beliefs that are targeted in the MCT-OCD, in order to identify the mode of action of all modules in greater depth. In contrast to the pilot study showing that patients’ mood worsened after the module on overestimation of threat/exaggerated sense of responsibility, patients’ mood did not worsen after any of the revised modules in the present study. Thus, the revision of the MCT-OCD can be regarded as successful in respect to the elimination of adverse effects.

Adverse effects of between-session effects of the pilot study (i.e., intensification of compulsions) were not evident in the revised MCT-OCD. This can be regarded as a further improvement. However, between-session changes contradicted our prior assumptions because no hypothesis-conformant result was found. Surprisingly, patients’ intolerance of uncertainty worsened (i.e., increased) the most one week after module #1 (false assumptions about OCD) compared to all other modules. Given that the module has a psychoeducational character, it might be assumed that patients developed a certain clarity about their symptoms and concluded that they were now more certain about particular concerns (e.g., the identification of their OC symptoms), and they therefore scored higher on the item that asked for confidence in choices. Thus, the module might either be slightly revised by conveying that some uncertainty will always remain and encourage patient acceptance of this, or the item that assessed this metacognition should be revised in order to assess it more precisely. However, the finding needs to be replicated in future studies in order to draw more valid conclusions.

The absence of other between-session effects is in line with our pilot study^14^, which did not show hypotheses-conformant results for between-session effects. Between-session effects could have been expected if the patients applied and practiced what they had learned from the session between sessions. However, the clinical impression was that patients often did not apply and practice what they had learned, which may have significantly reduced the between-session effects.

In addition to the several strengths of the present study (e.g., the specific analyses of session-specific effects in contrast to the overall efficacy of the MCT-OCD, secured diagnoses by trained raters in in-person assessments, revised intervention, and pre- and post-questionnaires), some limitations need to be acknowledged. First, the study and the therapy were conducted by the developers of the MCT-OCD (LJ, BH, FM), leading to a potential allegiance effect. Second, the between-session analyses may have been influenced by other concurrent outpatient therapies because patients were allowed to continue their treatment as usual. Third, the main analyses relied on self-report, which has several advantages (e.g., anonymity and thus possibly more honest feedback) but also comes with the disadvantage that items may have been misunderstood as well as the general disadvantages of self-report^15^. Forth, we did not explore whether changes in beliefs during the MCT-OCD are predictive of treatment outcome. This was not an aim of the current study, but it should be investigated in future studies. Fifth, no a priori power analysis was conducted for the present study. Sixth, single items were analysed in the present study and no quality criteria of the pre- and post-session questionnaires are available, which limits conclusions.

## Conclusions

The results of the present study could be used to inform another revision of the MCT-OCD. Future revisions could include revising module #1 (false assumptions about OCD) so that the increased intolerance of uncertainty one week after module #1 would be eliminated (see discussion on between-session effects). Notably, we now know that module #5 (control of thoughts) specifically targets patients’ need to control their thoughts (replicated result)^14^. Thus, the module can be considered successful in improving the need to control thoughts. Moreover, this module might be used not only within the scope of the MCT-OCD but also in individual therapy for patients who excessively control their thoughts. As mentioned above, homework should become mandatory in the MCT-OCD in order to strengthen the between-session effects as well as the transfer of the learning into patients’ everyday life. For future studies, the pre- and post-questionnaires need to be revised to include more items on other potential mechanisms of change in the different modules instead of only those items that are related to the topics of the modules. Based on the results of the present study, most modules (except for module #5) do not specifically improve the specific (meta)cognitive belief (e.g., perfectionism) that is targeted in the module but might act through something different (e.g., self-esteem). This needs to be explored in future studies. Based on our results, it is apparent that research on session-specific effects can be very fruitful for the evaluation of a therapy and the design of its revision as well as for the determination of the specific effects of the intervention or certain components of it. Using session-specific analyses instead or on top of the evaluation of the overall efficacy of an intervention can help to optimize therapies.

## Materials and methods

### Design

We conducted a randomized wait-list controlled trial. The study design (study protocol) of main trial have already been reported^15^. After baseline assessment (blinded rater), patients were randomized to participate either in MCT-OCD or in the wait-list control group and were assessed after eight weeks (post assessment). Both groups had access to care as usual. Informed consent was obtained from all patients, and the study was conducted in accordance with the Declaration of Helsinki. The study was approved by the Ethics Committee of the German Psychological Society (LJ112017) and was registered with the German Clinical Trials Register (Deutsches Register Klinischer Studien [DRKS]; DRKS-ID: DRKS00013539; registration date: 22/02/2018).

### Sample

Patients were screened for eligibility through a telephone interview. If they fulfilled the inclusion criteria, they were invited for a baseline in-person assessment and asked to complete a range of questionnaires. Patients were included if they were between 18 and 70 years of age, were willing to participate in the MCT-OCD, were suitable for outpatient group therapy (i.e., no acute suicidality), and had sufficient German language comprehension. Patients with current or lifetime psychotic symptoms, mania, a severe neurological disease, and/or current substance dependence were excluded. Due to recruitment difficulties, we broadened the inclusion and exclusion criteria to also include patients with a lifetime mood disorder with psychotic features, a lifetime high substance use disorder, or a neurological disease not associated with OC symptoms). For the present study, only patients who were randomized to the MCT-OCD, who participated in at least one MCT-OCD module, and who filled out at least one pre- and one post-session questionnaire were included in the analyses, resulting in a total sample of *N* = 34 for the present study.

### Metacognitive training

A psychotherapist in training (master’s degree in psychology) and a psychologist with a bachelor’s degree carried out the MCT-OCD. The sessions lasted approximately 90 min and were administered once a week over a period of 8 weeks. The group size was allowed to range between three and ten patients. The variation in the number of participating patients arose due to the open group format (i.e., patients were able to enter and drop of the group at any time). In the patient’s first session, they received a booklet with exercises for each session (designed to facilitate the transfer of the intervention into everyday life) and summaries of the most important content of the modules (exercises).

### Measures

The M.I.N.I. International Neuropsychiatric Interview 5th Ed. (M.I.N.I.; German version, 7.0.2)^16^ was used for the diagnosis of OCD and other comorbid disorders and to verify exclusion criteria. The gold standard for the assessment of patients’ OC symptom severity was used, the Yale-Brown Obsessive Compulsive Scale (Y-BOCS)^17^. Depressive symptom severity and frequency were assessed by a semi-structured interview, the Hamilton Depression Rating Scale (HDRS, 17-item version)^18^.

### Pre- and post-session questionnaires

The pre- and post-session questionnaires that were used in the pilot trial^14^ were revised in order to assess the (meta)cognitive beliefs that are targeted in the modules more specifically. Two items specific to each of the eight modules of the MCT-OCD (16 items in total) and three additional items assessing OC symptoms (obsessions and compulsions) and mood (see Supplementary material A for all items and the relevant abbreviations) were used, resulting in a total of 19 items, which were rated on a Likert scale (*1* = *fully disagree, 2* = *disagree, 3* = *not sure, 4* = *agree, 5* = *fully agree*). Patients filled out the pre-session questionnaire right before each session and the post-session questionnaire right after each session. To enable patients to provide their data under a pseudonym, each patient created an individual code that they used during all sessions.

### Strategy of data analysis

LMMs were calculated to analyze the main research questions of the present study due to the hierarchical structure of the data. Three different sets of calculations were run in order to identify (1) the amount of within-session changes for each module and each dependent variable, (2) the treatment effect over time for each dependent variable, and (3) the magnitude of between-session changes for each module. All analyses were run with all 19 dependent variables and thus all items of the pre- and post-session questionnaires.

The LMMs consisted of two levels: (1) repeated measures (pre- and post-session questionnaires) and (2) between-subject factor (i.e., the patients). For all analyses, a random intercept model and a random intercept, random slope model were calculated, and ANOVAs compared the two models in order to choose the model that provided the best fit. The decision on model fit was based on the Akaike information criterion (AIC): A significantly lower AIC indicated a better model fit^19^. If the random intercept, random slope model was used, it indicated a significant variability in the patients’ level of the specific variable at the start of the session and in the change within or between sessions. We decided to also test random slopes for the analyzed models by allowing the respective effect to vary between patients. For the approximation of parameters, the maximum-likelihood method was used^20^, and the beta values (*B*) represent the estimated effect sizes. In particular, *B* displays the magnitude of standard deviations of the changes due to the module under consideration compared to all other modules^21^. See Supplementary material B for the equations for all calculations and detailed information of the analyses and Supplementary material C for the AICs of each model. This strategy of data analysis was also used in two previous studies^14,22^. As exploratory analyses, we computed whether any other module had a stronger effect on the specific items compared to all the other modules. As we conducted exploratory analyses for the seven remaining modules, a Bonferroni-corrected alpha level of 0.05/7 = 0.007 was applied. Effect sizes of significant findings are presented in the results section and can be interpreted similar to the classification by Cohen^23^ (*d* = 0.2 small effect; *d* = 0.5 medium effect; *d* = 0.8 large effect).

### Power analyses

We used R package simr to estimate the power to find a certain effect size. According to the power analysis, the smallest effects that can be found for the used models (only insignificant results) and the sample of the present study with a power of greater than or equal to 0.80 range between small and moderate effects for the within-session analyses (0.39 ≤ *d* ≥ 0.70).

### Ethical standards

The authors assert that all procedures contributing to this work comply with the ethical standards of the relevant national and institutional committees on human experimentation and with the Helsinki Declaration of 1975, as revised in 2008. The authors assert that all procedures contributing to this work comply with the ethical standards of the relevant national and institutional guides on the care and use of laboratory animals.

## Supplementary information

Supplementary Information 1.

Supplementary Information 2.

Supplementary Information 3.

## Data Availability

The data that support the findings of this study are available from the corresponding author upon reasonable request.
