# Supplementary material for: Session-specific effects of the Metacognitive Group Training for Obsessive–Compulsive Disorder: significant results for thought control
Source: Sci Rep. 2020 Oct 20;10:17816. doi: 10.1038/s41598-020-73122-z (PMC7576173; doi:10.1038/s41598-020-73122-z)
Supplement: Supplementary file 1 — Supplementary Information 1. [file 41598_2020_73122_MOESM1_ESM.docx]

**Electronic Supplementary Material A**

| **Item** | **Abbreviation** |
| --- | --- |
| (1) It is important that I constantly observe my thoughts. | Thought monitoring |
| (2) I should be able to control my thoughts at all times. | Thought control |
| (3) Right now, I’m suffering from obsessive-compulsive thoughts. | Obsessions |
| (4) Right now, I’m suffering from compulsions. | Compulsions |
| (5) I’m in a bad mood right now. | Mood |
| (6) I am aware of the association of my obsessions and my compulsions. | Belief of being well informed about the disorder 1 |
| (7) I am aware of the effect of avoidance behavior on my compulsions. | Belief of being well informed about the disorder 2 |
| (8) Things are not right for me if they are not perfect. | Perfectionism 1 |
| (9) Making a mistake is as bad as completely failing. | Perfectionism 2 |
| (10) To feel safe, I have to be prepared for everything. | Intolerance of uncertainty 1 |
| (11) I have to be confident in my choices. | Intolerance of uncertainty 2 |
| (12) An immoral thought or impulse is as bad as its implementation. | Action fusion 1 |
| (13) Having violent thoughts means I cannot control myself. | Action fusion 2 |
| (14) Even though I am careful, I am convinced that something bad will happen. | Overestimation of Threat 1 |
| (15) A lot of things around me are dangerous. | Overestimation of Threat 2 |
| (16) If I do not act as if I am anticipating danger, I am responsible for any possible consequences. | Inflated sense of responsibility 1 |
| (17) If my actions can have even the slightest effect on an accident, then I am responsible for the result. | Inflated sense of responsibility 2 |
| (18) I associate many things in the environment with my compulsion. | Biased attention |
| (19) I often notice that I associate ordinary things with my compulsion. | Biased cognitive networks |
