# Supplementary material for: Session-specific effects of the Metacognitive Group Training for Obsessive–Compulsive Disorder: significant results for thought control
Source: Sci Rep. 2020 Oct 20;10:17816. doi: 10.1038/s41598-020-73122-z (PMC7576173; doi:10.1038/s41598-020-73122-z)
Supplement: Supplementary file 2 — Supplementary Information 2. [file 41598_2020_73122_MOESM2_ESM.docx]

**Electronic Supplementary Material B**

For the calculation of the demographic and psychopathological data, SPSS^®^ 25 (IBM Corp, 2017) was used, and for the main analyses, R (R Core Team, 2017) was used. The R lme4 package (Bates, Mächler, Bolker, & Walker, 2015) was used for the calculation of the models and the lmerTest package (Kuznetsova, Brockhoff, & Christensen, 2017) for the calculation of the *p*-values. Beta was divided by the standard deviation of the dependent variable in order to calculate effect sizes. Thus, the effect size indicates how many standard deviations the dependent variable changes within or between certain modules compared to all other modules.

For a more detailed understanding of the main calculations, the three equations of the models are presented here, which were run for all 19 dependent variables and all 8 modules. Equation (1) was used for the calculation of within-session changes. For a detailed description of the individual components of the equation, see below.

(1) ${dependent variable\_post}_{ti}$ = $\beta_{0}$ + $\beta_{1}$*${variable\_pre}_{ti}$ + $\beta_{2}$*${module}_{ti}$ + [$u_{0i}$ + $(u_{2i}$)+ $e_{ti}$]

- ${dependent variable\_post}_{ti}$ = score of the dependent variable after a session
- $\beta_{0}$= mean score of the dependent variable after sessions in which the investigated module was not provided
- $\beta_{1}$ = mean change in the dependent variable for each session
- ${module}_{ti}$= each module was either coded with a 1 (the session in which patient *i* completed the module under consideration) or was coded with a 0 (all other sessions)
- $\beta_{1}$*${variable\_pre}_{ti}$ = differences in scores of the dependent variable before the session
- $\beta_{2}$ = mean difference score in symptoms after the session in which the module under consideration was provided compared to all other sessions in which the module was not provided
- $u_{2i}$ = predicts within-subject variance for testing the random effects

Equation (2) was used for the calculation of the change in the dependent variable over the duration of the treatment. The individual components of the equations are detailed below.

$(2) {Dependent variable}_{ti}$ = $\beta_{0}$ + $\beta_{1}$*${session}_{ti}$ + [$u_{0i}$ + $u_{1i}$ + $e_{ti}$]

- ${dependent variable}_{ti}$ = the patient’s *i* score in the dependent variable in session *t*
- $\beta_{0}$ = mean dependent variable score before the first session across all patients
- ${session}_{ti}$ = the session variable was centered at 1 in order to allow the calculation from the first to the last session
- $u_{0i}$ = variation between patients in terms of the score of the dependent variable before the treatment began
- $u_{1i}$ = variance in individual change rates
- $e_{ti}$ = session-specific error term

Equation (3) was used for the calculation of the change between sessions. The components of the equation not already explained are displayed in detail below.

(3) ${Dependent variable\_pre}_{t+1i}$ = $\beta_{0}$ + $\beta_{1}$*${variable\_post}_{ti}$ + $\beta_{2}$*${module}_{ti}$ + [$u_{0i}$ + $(u_{2i}$)+ $e_{ti}$]

- ${Dependent variable\_pre}_{t+1i}$= pre variable score after the previous week’s session
- $\beta_{1}$*${variable\_post}_{ti}$ = difference in symptom scores after the session
