# Supplementary material for: Session-specific effects of the Metacognitive Group Training for Obsessive–Compulsive Disorder: significant results for thought control
Source: Sci Rep. 2020 Oct 20;10:17816. doi: 10.1038/s41598-020-73122-z (PMC7576173; doi:10.1038/s41598-020-73122-z)
Supplement: Supplementary file 3 — Supplementary Information 3. [file 41598_2020_73122_MOESM3_ESM.docx]

**Electronic Supplementary Material C**

| Models that analyzed the change over the duration of the treatment. | | |
| --- | --- | --- |
| Variable | AIC of the random intercept model | AIC of the random intercept, random slope model |
| Thought monitoring | 505.84 | 506.37 |
| Thought control | 504.39 | 506.83 |
| Obsessions | 584.24 | 587.46 |
| Compulsions | 493.51 | 493.90 |
| Mood | 548.96 | 551.22 |
| Belief about being well informed about OCD 1 | 439.21 | 433.65 |
| Belief about being well informed about OCD 2 | 410.8 | 397.4 |
| Perfectionism 1 | 424.21 | 428.17 |
| Perfectionism 2 | 538.30 | 537.03 |
| Intolerance of uncertainty 1 | 432.39 | 434.58 |
| Intolerance of uncertainty 2 | 316.34 | 314.30 |
| Action fusion 1 | 461.96 | 452.48 |
| Action fusion 2 | 475.56 | 470.56 |
| Overestimation of Threat 1 | 467.48 | 456.44 |
| Overestimation of Threat 2 | 411.98 | 415.71 |
| Inflated sense of responsibility 1 | 504.46 | 504.69 |
| Inflated sense of responsibility 2 | 499.22 | 482.25 |
| Biased attention | 513.37 | 507.71 |
| Biased cognitive networks | 455.80 | 456.62 |

| Models that analyzed within-session effects. | | |
| --- | --- | --- |
| Variable | AIC of the random intercept model | AIC of the random intercept, random slope model |
| Thought monitoring  Modul 1  Modul 2  Modul 3  Modul 4  Modul 5  Modul 6  Modul 7  Modul 8 | 415.18  415.12  412.23  415.02  414.96  414.29  415.01  413.73 | 417.74  418.82  415.14  408.73  418.41  418.05  418.14  415.39 |
| Thought control  Modul 1  Modul 2  Modul 3  Modul 4  Modul 5  Modul 6  Modul 7  Modul 8 | 464.34  462.98  462.01  465.13  455.98  463.82  464.90  465.38 | 468.32  464.36  465.20  469.05  457.92  465.75  468.89  469.20 |
| Obsessions  Modul 1  Modul 2  Modul 3  Modul 4  Modul 5  Modul 6  Modul 7  Modul 8 | 484.34  484.23  479.03  484.66  484.71  484.82  484.67  482.29 | 486.95  478.74  482.26  484.96  487.15  488.27  488.57  486.29 |
| Compulsions  Modul 1  Modul 2  Modul 3  Modul 4  Modul 5  Modul 6  Modul 7  Modul 8 | 389.56  388.46  389.56  389.42  389.54  388.59  389.45  389.01 | 380.99  392.37  393.47  392.12  392.23  392.55  390.74  392.89 |
| Mood  Modul 1  Modul 2  Modul 3  Modul 4  Modul 5  Modul 6  Modul 7  Modul 8 | 474.62  473.50  472.08  474.86  469.20  474.91  474.99  473.83 | 475.74  475.15  475.24  478.85  473.08  478.82  478.97  477.70 |
| Belief about being well informed about OCD 1  Modul 1  Modul 2  Modul 3  Modul 4  Modul 5  Modul 6  Modul 7  Modul 8 | 349.28  334.79  348.06  348.07  348.02  349.23  349.03  349.09 | 346.84  329.94  351.88  350.01  351.93  352.74  352.77  350.82 |
| Belief about being well informed about OCD 2  Modul 1  Modul 2  Modul 3  Modul 4  Modul 5  Modul 6  Modul 7  Modul 8 | 276.22  277.02  274.91  276.54  277.14  277.04  277.15  276.36 | 279.72  277.91  278.47  279.37  268.40  279.90  280.78  271.57 |
| Perfectionism 1  Modul 1  Modul 2  Modul 3  Modul 4  Modul 5  Modul 6  Modul 7  Modul 8 | 405.57  407.38  406.01  406.80  407.44  402.90  407.35  406.98 | 402.25  411.38  409.41  410.74  411.27  406.42  411.27  410.29 |
| Perfectionism 2  Modul 1  Modul 2  Modul 3  Modul 4  Modul 5  Modul 6  Modul 7  Modul 8 | 443.94  444.71  445.13  444.58  443.06  445.05  445.09  444.22 | 447.06  448.69  448.97  448.05  446.75  448.59  449.03  447.21 |
| Intolerance of uncertainty 1  Modul 1  Modul 2  Modul 3  Modul 4  Modul 5  Modul 6  Modul 7  Modul 8 | 446.13  446.66  447.38  446.82  446.70  445.6  444.70  445.46 | 442.78  450.58  450.84  450.73  450.21  448.8  443.35  447.98 |
| Intolerance of uncertainty 2  Modul 1  Modul 2  Modul 3  Modul 4  Modul 5  Modul 6  Modul 7  Modul 8 | 335.36  334.14  335.35  335.49  332.19  335.40  335.24  334.69 | 335.37  337.43  337.87  339.14  331.17  338.03  331.79  323.67 |
| Action fusion 1  Modul 1  Modul 2  Modul 3  Modul 4  Modul 5  Modul 6  Modul 7  Modul 8 | 369.60  368.68  369.52  369.42  366.71  369.45  369.20  369.27 | 371.02  368.04  373.43  372.45  369.55  369.81  373.13  372.77 |
| Action fusion 2  Modul 1  Modul 2  Modul 3  Modul 4  Modul 5  Modul 6  Modul 7  Modul 8 | 411.51  411.40  411.56  408.51  407.81  411.62  411.14  409.41 | 414.44  415.06  414.84  412.37  411.36  414.50  414.53  408.45 |
| Overestimation of Threat 1  Modul 1  Modul 2  Modul 3  Modul 4  Modul 5  Modul 6  Modul 7  Modul 8 | 422.46  423.05  423.36  420.41  422.00  423.74  423.73  419.96 | 425.65  426.66  425.04  423.81  426.00  427.68  427.70  423.60 |
| Overestimation of Threat 2  Modul 1  Modul 2  Modul 3  Modul 4  Modul 5  Modul 6  Modul 7  Modul 8 | 414.19  411.55  416.17  415.12  416.38  412.65  415.16  415.57 | 417.50  415.05  418.78  415.98  420.17  416.15  418.86  418.73 |
| Inflated sense of responsibility 1  Modul 1  Modul 2  Modul 3  Modul 4  Modul 5  Modul 6  Modul 7  Modul 8 | 385.37  384.69  385.25  383.81  384.69  385.42  384.17  385.41 | 389.34  385.99  389.22  385.67  388.40  389.42  387.39  388.93 |
| Inflated sense of responsibility 2  Modul 1  Modul 2  Modul 3  Modul 4  Modul 5  Modul 6  Modul 7  Modul 8 | 390.78  390.53  390.79  389.52  390.78  390.81  390.28  389.60 | 375.85  393.85  389.40  393.37  394.65  394.56  393.54  393.59 |
| Biased attention  Modul 1  Modul 2  Modul 3  Modul 4  Modul 5  Modul 6  Modul 7  Modul 8 | 406.05  405.32  407.39  407.30  407.96  402.42  407.95  406.88 | 409.99  408.49  408.62  411.23  411.08  405.96  411.95  410.82 |
| Biased cognitive networks  Modul 1  Modul 2  Modul 3  Modul 4  Modul 5  Modul 6  Modul 7  Modul 8 | 409.86  410.43  409.28  409.64  409.37  410.18  410.45  409.80 | 414.13  414.29  411.24  412.70  405.68  413.77  414.44  409.57 |

| Models that analyzed between-session effects. | | |
| --- | --- | --- |
| Variable | AIC of the random intercept model | AIC of the random intercept, random slope model |
| Thought monitoring  Modul 1  Modul 2  Modul 3  Modul 4  Modul 5  Modul 6  Modul 7  Modul 8 | 399.02  398.54  399.04  398.98  398.99  398.54  397.57  399.04 | 401.77  402.40  396.75  401.57  402.56  401.85  398.51  402.37 |
| Thought control  Modul 1  Modul 2  Modul 3  Modul 4  Modul 5  Modul 6  Modul 7  Modul 8 | 419.51  419.10  419.49  419.54  418.82  418.70  419.26  418.59 | 423.28  419.73  419.32  420.92  422.40  422.14  423.25  416.56 |
| Obsessions  Modul 1  Modul 2  Modul 3  Modul 4  Modul 5  Modul 6  Modul 7  Modul 8 | 450.23  450.63  449.35  451.21  450.79  449.46  450.59  451.23 | 453.93  454.34  452.23  454.95  452.62  452.70  453.63  455.24 |
| Compulsions  Modul 1  Modul 2  Modul 3  Modul 4  Modul 5  Modul 6  Modul 7  Modul 8 | 390.62  387.61  389.80  391.08  389.77  391.09  391.04  390.18 | 392.60  390.55  393.80  395.08  393.72  395.03  390.58  391.59 |
| Mood  Modul 1  Modul 2  Modul 3  Modul 4  Modul 5  Modul 6  Modul 7  Modul 8 | 431.39  431.96  430.15  432.03  430.71  430.77  431.67  431.62 | 433.52  435.08  432.27  436.02  434.67  434.77  435.63  435.12 |
| Belief about being well informed about OCD 1  Modul 1  Modul 2  Modul 3  Modul 4  Modul 5  Modul 6  Modul 7  Modul 8 | 292.27  291.40  292.67  292.00  292.65  292.32  292.08  292.34 | 288.55  294.29  296.66  295.87  296.34  295.19  294.44  291.38 |
| Belief about being well informed about OCD 2  Modul 1  Modul 2  Modul 3  Modul 4  Modul 5  Modul 6  Modul 7  Modul 8 | 310.84  310.76  311.74  311.93  310.79  310.58  310.35  311.76 | 313.92  313.28  314.82  315.25  313.72  313.27  313.98  312.05 |
| Perfectionism 1  Modul 1  Modul 2  Modul 3  Modul 4  Modul 5  Modul 6  Modul 7  Modul 8 | 359.90  364.98  365.53  365.69  364.80  365.69  365.68  365.57 | 358.07  368.98  369.47  369.69  368.45  369.69  369.68  362.30 |
| Perfectionism 2  Modul 1  Modul 2  Modul 3  Modul 4  Modul 5  Modul 6  Modul 7  Modul 8 | 421.45  422.74  421.97  422.64  422.81  421.64  422.76  422.60 | 424.96  420.56  425.96  426.64  425.66  425.63  426.76  426.39 |
| Intolerance of uncertainty 1  Modul 1  Modul 2  Modul 3  Modul 4  Modul 5  Modul 6  Modul 7  Modul 8 | 347.78  349.07  348.96  349.76  349.36  349.71  349.71  349.69 | 349.78  353.02  345.81  345.17  350.54  351.43  353.59  349.65 |
| Intolerance of uncertainty 2  Modul 1  Modul 2  Modul 3  Modul 4  Modul 5  Modul 6  Modul 7  Modul 8 | 213.04  222.08  223.59  221.10  220.95  223.37  223.64  218.04 | 216.76  222.18  226.84  222.63  224.93  224.44  227.44  211.08 |
| Action fusion 1  Modul 1  Modul 2  Modul 3  Modul 4  Modul 5  Modul 6  Modul 7  Modul 8 | 341.48  341.55  342.13  341.93  342.19  339.53  342.15  341.85 | 345.48  343.08  346.13  344.26  346.19  343.53  341.38  344.33 |
| Action fusion 2  Modul 1  Modul 2  Modul 3  Modul 4  Modul 5  Modul 6  Modul 7  Modul 8 | 363.73  367.40  366.50  367.04  364.95  365.72  366.96  367.33 | 364.27  368.40  361.71  370.66  368.54  369.31  370.86  371.26 |
| Overestimation of Threat 1  Modul 1  Modul 2  Modul 3  Modul 4  Modul 5  Modul 6  Modul 7  Modul 8 | 364.55  364.70  364.77  364.92  364.89  364.90  362.71  364.24 | 367.88  365.55  368.76  368.82  368.86  367.48  366.66  360.11 |
| Overestimation of Threat 2  Modul 1  Modul 2  Modul 3  Modul 4  Modul 5  Modul 6  Modul 7  Modul 8 | 305.70  299.25  305.08  305.96  306.13  304.71  303.30  305.99 | 309.27  303.07  308.03  306.35  303.41  306.93  304.05  304.63 |
| Inflated sense of responsibility 1  Modul 1  Modul 2  Modul 3  Modul 4  Modul 5  Modul 6  Modul 7  Modul 8 | 370.82  370.78  370.37  370.15  370.64  367.53  371.13  368.98 | 374.62  374.04  374.06  374.15  372.40  369.20  375.06  362.43 |
| Inflated sense of responsibility 2  Modul 1  Modul 2  Modul 3  Modul 4  Modul 5  Modul 6  Modul 7  Modul 8 | 372.20  369.19  372.12  367.04  370.33  369.22  372.20  371.89 | 375.96  369.18  376.03  369.95  369.33  372.27  376.11  373.07 |
| Biased attention  Modul 1  Modul 2  Modul 3  Modul 4  Modul 5  Modul 6  Modul 7  Modul 8 | 364.05  363.97  362.62  364.01  363.38  362.32  363.73  362.79 | 367.47  360.81  366.47  367.77  367.20  366.12  367.23  362.57 |
| Biased cognitive networks  Modul 1  Modul 2  Modul 3  Modul 4  Modul 5  Modul 6  Modul 7  Modul 8 | 343.83  344.93  344.06  344.50  344.91  339.13  344.58  344.19 | 347.79  345.61  346.94  348.28  348.89  340.41  348.35  343.80 |
